# The Hospital Nursing Supplement

**Published:** 1893-02-11

**Authors:** 


### "The Hospital" Nursing Mirror.

Being the Extra Nursing Supplement of "The Hospital" Newspaper.

[Contributions for this Supplement should be addressed to the Editor, The Hospital, 140, Strand, London, W.C., and should have the word "Nursing" plainly written in left-hand top corner of the envelope.]

### News from the Nursing World.

### LITTLE INVALIDS.

Soon after Easter it is proposed to give a theatrical performance in aid of the funds of that ideal society, the Invalid Children's Aid Association, which has its headquarters at 18, Buckingham Street. Their Royal Highnesses Princess Mary of Teck and her daughter have promised their patronage; the latter, Princess May, being President of the I.C.A.A.

### THE LITTLE KING.

The young King of Spain is suffering from a mild attack of scarlet fever, and it is pleasant to hear that he begins to make satisfactory progress towards convalescence. His Royal mother and his own English nurse are in constant attendance on little King Alphonso. The Queen has thoughtfully dispensed with the attendance of those ladies who, having children of their own, would run risks of conveying infection to the latter if their duties at Court were not thus considerately remitted.

### A PLEASANT ENTERTAINMENT.

Previous to her departure from the National Hospital, Queen Square, Miss Hadley-Scott, Staff-Nurse, gave a pleasant entertainment to the patients in Morgan ward. The matron and sisters were present, and as many of the nurses as could be spared from their duties were also of the party. The Chaplain made an excellent president, and some good songs were contributed by members of the medical staff and also by the patients. Several of the latter could not leave their wheel-chairs, but their powerlessness in that respect certainly seemed in no way to have injured the quality of unusually beautiful voices. Glees were pleasantly rendered by the matron and some of the nurses. Before "God Save the Queen" signalled the close of the entertainment, the Chaplain, in a few well-chosen words, expressed the universal regret of all those assembled at the departure of the lady who had discharged her duties so faithfully, cheerfully, and patiently. A pleasant surprise was provided for their nurse by the patients themselves in the form of a set of beautiful silver links, with monogram, and a brooch to match. These were modestly presented to her at the end of the evening, with a card which bore a grateful and affectionate inscription.

### THE BOURNEMOUTH POOR.

The annual meeting of the Society for Providing Nurses for the Sick Poor at Bournemouth took place recently. One of the speakers referred to the fact that poverty was rather out of sight in their town. The visitors who keep to the popular public walks have little knowledge of the large and increasing districts inhabited by a very poor class of people. There has been a great demand for the services of the nurses, and two additional ones have been engaged for six months, making four in all. They are each of them fully employed, and the Committee appeal urgently for funds to enable them to retain the extra nurses permanently. The speeches made at the meeting were particularly pleasant ones, and it was gratifying to hear the universal opinions as to the value of trained nurses, and also the specially hearty appreciation of these workers at Bournemouth. Many rich invalids go to this fashionable health resort, and their recovered strength often dates from their visits. Surely no better thank-offering could be devised than a cheque handed to an association which provides good attendance for the sick poor, to whom health is as essential as it is to the prosperous. The latter have secured and been thankful for skilled nursing in their turn, and can reasonably be asked to provide it for their poorer brethren.

### UNWATCHED DEATH-BEDS.

The action recently taken by the Local Government Board with regard to the nursing at the Eastville Workhouse is certainly highly commendable. The letter addressed by them to the Barton-Regis Board of Guardians was read at the weekly meeting. Its request that the remarks of their medical officer, Dr. Downes, should receive the attention of the guardians was courteously complied with. After an inspection of the workhouse Dr. Downes made a report, from which the following is an extract: "The paid nursing staff is not such as may now be looked for in the wards of so large an infirmary. . . There is no paid night nurse in the general sick wards, although the average number of deaths in the workhouse is 170 yearly." The resident medical officer, Dr. Bernard, also remarked that the insane inmates "had not nearly enough attendants." Miss Woollam wisely urged that the matter should be gone into thoroughly, and not only insisted on the necessity for night nurses, but also on their being efficient ones. She remarked that "many of the deaths which had been referred to by Dr. Downes occurred at night," and a more pathetically significant sentence, coming after the confession that night nurses were not employed, could hardly be conceived. Another lady guardian seconded the resolution that two night nurses should be appointed in the infirmary wards as soon as possible. It was decided that a committee should inquire into the matter, and report on it to the Board, therefore the sick paupers at Eastville may have reasonable hopes of more satisfactory attendance in future.

### MRS. OR MISS?

The Langport Board of Guardians held a meeting lately which had several amusing features about it. They wished to select a nurse for the workhouse, and they were fortunate enough to have four candidates for this responsible post. The testimonials were all read, and the choice lay between Mrs. Babbage and Miss Coleman. Unluckily, Langport does not appear to possess any lady guardians, otherwise a proposal to engage one of the applicants without a personal interview would assuredly never have been seconded. Mr.

Wyatt wisely objected to any such proceeding, and rightly refused to countenance the appointment of anyone who had not been previously seen by the Board. The Rev. G. Smith, who was one of the advocates for an immediate decision, gave it as his opinion that forty was a suitable age, rather than twenty-six. He had evidently made up his mind that a "Miss" must be "a young person," and a "Mrs." of necessity "a staid person." Mr. Wyatt said "a staid person might be seventy." This certainly would be a little too mature an age for an attendant for sick persons. However, thanks to the common sense of Mr. Wyatt and Mr. Knight, it was decided by a small majority to invite the two candidates to appear before a committee appointed by the Board to decide on the fitness of "Mrs." or "Miss."

### PROGRESS AT DUNDEE.

The new parochial hospital at Dundee is to have its nursing staff formed after the intelligent methods approved by modern authorities. The head nurse, we presume a trained and qualified lady, capable of teaching her subordinates, will be assisted by eight charge nurses and four probationers. Probably the board will soon discover that they can advantageously double the number of the latter. In the reports of the meeting where this new important departure was resolved upon no mention is made as to the probable age of candidates to be chosen for the various posts. Doubtless, however, this question has been well considered, and the new hospital will be in no way behind those established institutions which have universally accepted twenty-five as the lowest desirable age for young women to enter on the serious profession of trained nursing.

### MELBOURNE NURSES.

The Melbourne hospitals are quite unanimous on one subject, and that is the sadly-familiar "want of funds." The Austin Hospital, says *The Argus*, has found its resources severely taxed in consequence of the opening of a new wing for the reception of consumptive patients, and nearly all the charities—and they form a surprisingly long list—have suffered from the commercial depression of the country. However, the cordial appreciation by all classes of the work done by the Melbourne District Nursing Society encourages a hope that the nurses' labours will be in no way curtailed. The association provides for the sick poor the advantages of trained attendance in their own homes. During the last six months of 1892 the nurses paid 9,518 visits to 404 patients, doing such service as it is impossible entirely to estimate. Their efforts on behalf of a class of sufferers who sorely need intelligent help, must give them a claim for support, whether "times" are owned to be "pretty good" or "very bad" in the colony.

### NURSES AT VANCOUVER.

Sympathy and help are always urgently needed by the nurses in Vancouver. Justly enough they found claims to assistance for the aid which is constantly granted by them to sick and impecunious English men and women, who find their way to British Columbia just as surely as the rich and prosperous "globe-trotter" does. Sister Francis and her nurses tried to forget their financial anxieties at Christmas, and succeeded in giving pleasure to many past as well as present patients. All the provisions for the feast were contributed by the liberality of various friends; and on the evening of the grand entertainment at the Home, the Electric Tramcar Company conveyed the guests gratuitiously in carriages specially run for this purpose. The decorations consisted of many handsome flags, which were lent by the officers of the s.s. *Empress of India*, then in the harbour. The festivity made a pleasant break in the busy lives of the workers, and a grand white-washing and colouring of the wards have since taken place in preparation for another year's labours. Sister Francis gratefully acknowledges gifts of clothing and children's garments from Mrs. W. Goslett, Miss Routledge, Miss Keiller, and Miss Greengrass. Nurse Gilchrist, of the City Hospital, Edinburgh, sent thirty-six shillings, which she had kindly collected. Sister Francis will be very glad to acknowledge many more such gifts to further the good nursing at St. Luke's, Vancouver.

### NURSES' HOME, BOSTON, U.S.A.

When the new maternity building was completed at the New England Hospital for Women and Children at Boston, accommodation for the nursing staff was rapidly secured. The old maternity block was transformed into an admirable Home. The walls and ceilings were cleansed and painted, and new floors were laid down, and all arrangements needed for the comfort of the twenty nurses who comprise the staff were satisfactorily accomplished.

### NATIVE WHIMS.

In India the natives are often prejudiced against poultices, and it is not unusual for a patient ill with pneumonia, on whom the nurse has carefully adjusted one of these applications, to be found shortly afterwards sitting on the floor, without clothes, because "he felt hot," the poultice being found carefully hidden away, probably amongst the discarded blankets. Fomentations are much preferred by the natives, and they like having these applied to all parts of the body. Linseed is so dear in the hill stations that flour is often used as a substitute.

### SHORT ITEMS.

It has been decided to establish a staff of private nurses at Hereford. They will reside at the Infirmary between their cases, and be under the control of the matron. The fees suggested are moderate ones; and although it is hoped that the scheme will prove self-supporting, no profit will be made out of it by the Infirmary.—The Workhouse Nursing Association has appointed a staff of trained nurses to seven additional union infirmaries during the past year. There are now 33 boards which rely entirely on the Board for their supply of nurses. Trained matrons are still the exception in infirmaries; but wherever skilled nurses hold these important posts they appear to do excellent work, which is highly appreciated.

### "THE HOSPITAL" ENDOWED BED.

Subscriptions for the Nurses Bed are now due. All contributions sent to The Editor, The Lodge, Porchester Square, will appear in The Hospital. We have pleasure in acknowledging the following subscriptions: Miss Goulton, £2 2s., collected; Mrs. Creighton Hale, £1 1s.; Burton-on-Trent Institute, 6s.; Nurses Annie, 2s. 6d.; E. Bishop, 1s. 6d.; F. M. Shore, 1s. 6d.; Elms, 2s. 6d.; W. P., £1; Dagmar, 5s.; T. H. Nevill, £2 2s. Making total amount received up to date, £9 7s. 6d.

### A DISABLED NURSE.

The following donations have been received during the week, in company with many kind expressions of sympathy for the disabled nurse: R. N., 2s. 6d.; Putney, 788, 5s.; E. M. Turner, 5s.; M. Quinn, 2s. 6d.; M. Hunt, 2s. 6d.; Anon, 3s.; E. R. D., 2s.; H. N., 2s. 6d.; Nurses Spoor and Dodds, 5s.; Nurse Beal, 2s. 6d. Making total amount received up to date, £8 5s.

### Management of Consumptives.

### I.—GENERAL DESCRIPTION OF CONSUMPTION.

In giving a general account of consumption, much of importance on the subject will of necessity have to be left untouched owing to want of space, and to the fact that it does not directly concern the nurse to know it. Only so much of the disease as should be understood by anyone who is nursing consumption will be given here. Let us first consider the pathology and morbid anatomy of the disease. It will be well before doing this to define exactly what we mean by consumption. It must be clearly understood that in these papers, when we speak of consumption, we mean that disease which is primarily due to the tubercle bacillus or its product, in other words, tubercular consumption. Other names for this are tubercular phthisis, or tuberculosis of the lungs. Tubercular consumption then may be defined as a disease of the lungs of which the primary cause is supposed to be the tubercle bacillus, a disease in which destruction of the lung substance takes place by a process of ulceration. Now, when a person who is predisposed, from some cause or other, to take consumption, inhales a number of tubercle bacilli, the result is that, owing to the irritating action of these germs or their products, a number of small, grey rounded bodies, about the size of a millet seed, begin to form in the lung. These bodies are called tubercles. At first they are scattered about chiefly at the apex of the lung; but soon they coalesce, and a patch of varying size is formed. Now these small tubercles have no blood supply of their own, so that they soon grow out of all proportion to their supply of nutriment; the result of the want of food, so to speak, of these tubercles is that they very soon begin to show signs of decay and ultimately die. If one looks at tubercles in a more advanced state, there can generally be seen a minute yellow spot in their centres, the tubercle is beginning to soften. When a large patch of tubercles, which have become closely packed together, begin to soften and break down, the debris is coughed up in the form of purulent expectoration, and very soon what was a large patch of tuberculous deposit, becomes converted into cavity, and the patient is then said to have consumption which has gone on to excavation. Thus, by the deposition in the lung of the tubercle, its substance is destroyed and a cavity is the result. Around a patch of tuberculous matter there is a zone of inflammation. The wall of the cavity when first formed is thin, rough, and uneven, and secretes a large amount of pus; as it gets older, its wall becomes thick and smooth, and very little secretion takes place from it. We have already said that the lung tissue itself is destroyed, and when once destroyed it can never be reproduced again. As the cavity gets older it contracts and becomes smaller and smaller, until, if the patient lives long enough, all that can be seen of it is a scar of white fibrous tissue; the cavity is then said to be healed. While all this has been going on in the lung the pleura, which covers it, has also become inflamed, thickened, and adherent to the adjacent layer in the chest wall. During the progress of the lung disease the tubercular process may be going on in other parts of the body, the most frequent localities being the larynx, the intestines, the peritoneum, and the membranes covering the brain. Sometimes every organ almost in the body is studded with these small tubercles, and the case is then said to be one of general miliary tuberculosis. When the larynx becomes affected, tubercles are deposited on the vocal chords and other parts of that organ. They soon begin to break down in the manner described, and the patient loses his voice, suffers great pain, and is unable to swallow his food, or cough. The tubercle in the larynx is generally supposed to be caused by infection from the lungs by means of the expectoration continually passing through it. Similarly there are good grounds for supposing that many of the cases of tuberculosis of the intestine are due to the swallowing of the expectoration, instead of coughing it up. When this does take place the small intestine and the beginning of the large intestine are spotted with ulcers, some as large as a shilling, others larger, whilst others are not so large. These ulcers are due to the same general cause, viz., breaking down of tubercles which have been deposited there. The outside of the bowel which is covered by a smaller membrane, called the peritoneum, participates in this change, and sometimes the whole peritoneal surface is studded with tubercles. The case is then said to have tubercular peritonitis. When ulceration of the intestine takes place diarrhœa is the result. The intestines being so frequently affected with tubercle, it is a cause of wonder that the stomach is the last place in the body in which one would expect to find a tubercular ulcer; indeed, there is no indubitable case on record of such an occurrence. When the membranes of the brain are affected with tubercle the case is then one of tubercular meningitis. The patient with this complication complains of headache, becomes more and more drowsy, until at last he is quite unconscious, and very soon dies.

These, then, are the main points in the morbid anatomy of consumption and its complications. Summarising them, we may say that the tubercle bacillus or its products, is the cause of the disease; that by irritation tubercles are formed in the lungs; that these tubercles, from their peculiar constitution, must very soon of necessity soften and break down; that these tubercles coalesce, forming a cheesy patch; that when this patch of tuberculous matter breaks down a cavity is formed; that the cavity tends to heal and contract; that the apices of the lungs are the common situation for tubercles; and lastly, that other organs in the body, of which the chief are the intestines, the larynx, the peritoneum, and the membranes of the brain, become also the seat of tubercles.

*(To be continued.)*

### The Nurses' Bookshelf.

"THE Hospital Service Book" (*Henry Frowde, Amen Corner*), by the Rev. C. Parkhurst Baxter, contains "short services for use in the wards of hospitals and infirmaries, with the office for Holy Communion, and simple prayer for communicants, taken chiefly from the Book of Common Prayer." The little volume seems admirably adapted for the purposes above stated, for it possesses the great merit of containing all that is necessary in good print and with plain directions, yet without being "overloaded" like so many manuals already provided for pastoral visitation, and for the use of invalids. The hymns have been very carefully selected, and the services are so thoroughly liturgical that, we venture to think, the book will commend itself to all schools of thought, at least within the Church. The Bishop of Southwark, in a letter printed next to the preface, says that it provides "A simple companiom to the Holy Communion, not over-burdened with directions, nor advising, during sickness, too much self-analysis, but with sufficient guidance to make the instructions of the office clear, and to serve as a preliminary to further devotions when health is restored." As such the book may be used by the clergy in all parochial visitations of the sick. We believe that as it becomes known its uses will by no means be confined to hospitals.

### Examination Question for March.

"EXPLAIN the duties and responsibilities of a nurse in charge of cholera patients."

Answers must be sent in by March 1st. They must be concise, but thorough, written on one side of the paper only, accompanied by writer's name and address, and directed to "Nursing," Editor of THE HOSPITAL.

### Requisites for Nurses.

Messrs. Allen and Hanbury's "Personal Equipments for Nurses" are both varied and valuable; in fact, a visitor at their establishment in Plough Court, Lombard Street, becomes speedily impressed by the large number of admirable appliances of all kinds which are in readiness to facilitate nurses' labours in each separate branch of their profession. For the district nurse there are bags of many kinds at different prices. Some are fitted with outside and inside pockets, some are of leather, others of morocco, leather lined. They can be purchased with contents complete, as stated in Messrs. Allen and Hanburys instructive catalogue, or the bag only may be selected, and the articles desired can be chosen by the nurse whom experience has taught the special requirements of her own daily work. The number of things which are packed, after a convenient and ingenious method, into these bags is little short of miraculous. It hardly seems respectful to talk of conjuring tricks in connection with so serious a calling as that of a nurse, and yet the words "sleight of hand" come unbidden when a preliminary investigation of the contents of one of these moderate-sized hand-bags takes place. From a bath thermometer to tape and thread, from minim measure to bandages and disinfectants, nothing is omitted and much is remembered which it is not easy to bear in mind until the emergency is present which calls urgently for the administration of immediate remedies. Chatelaine, wallet, and pocket cases can be obtained, and the second and third of these are supplied in a combination form if preferred. The simplest wallet—of morocco with two leather straps to fasten it securely to the waistband—is a very convenient and certainly a very cheap case for hospital workers. It is well arranged for avoiding any of that jangling which is such an objectionable accompaniment to a nurse's progress through the ward or sick-room. Some of the wallets and pocket cases contain a larger supply of instruments, and are therefore specially suited for country nurses or for workers in distant colonies. Some cases are made of lizard leather, which is more expensive than the familiar and serviceable morocco. "Hypoderm cases," of aluminium or morocco, contain beautiful little English-made syringes and their accessories; and clinical thermometers of every kind are kept in stock. Obstetric nurses can obtain the most approved forms of belts or binders, and Messrs. Allen and Hanbury have recently completed one of the latter which has many advantages. It is known as the "Nurse Ellen Binder," and has two triangular elastic gussets at the top, giving expansion and permitting the band to be considerably higher than would otherwise be feasible. Two additional straps of india-rubber tubing or flannel are special characteristics as well as the gussets of the "Nurse Ellen," and the binder has certainly several other advantages which would further the comfort of a patient as well as the convenience of a nurse. The chart book designed by this firm is a particularly convenient one for size, simplicity, and also for economy, three essentials in private and district nursing. The "Ambulance Station Equipment" forms almost a separate department, it is such an apparently elaborate yet practically such a simple one. The various dressings are packed in an excellent antiseptic fashion which cannot be too highly commended. A busy district nurse needs all aids of this kind to enable her, in the midst of many drawbacks and difficulties, to carry out conscientiously the essential principles imbued during her own surgical training.

### London Obstetric Society.

Nurse Marie Pickering and Nurse Jean Herriott, who trained at the Manchester Maternity Hospital, have obtained the diploma of the L.O.S.

### The Nurses' Co-operation.

The second annual meeting of the Nurses' Co-operation took place at 20, Hanover Square, on February 2nd, and was very well attended in spite of the unpropitious weather. It was impossible to doubt the genuine interest and sympathy with which the audience regarded the association, whose objects were graphically described by the various speakers. Sir W. MacCormac was in the chair, and amongst those present were Dr. Goodhart, General Ross, Dr. Hadden (Hon. Treasurer), Mr. Bernard Pitts, General Swanston, Mr. Charles Cheston, Miss R. Pritchard. Dr. and Miss Napper, Mr. Morten, Miss Honnor Morten, Mr. Slaughter, Mr. Nicholson, Mr. and Mrs. Ll. Roberts, Miss Elms, Miss Florence Routledge, Miss Belcher, and many others. Mr. Michelli read letters regretting their unavoidable absence from H.R.H. Princess Mary of Teck (Patron of the Nurses' Co operation), the Duchess of Portland, Lord Sandhurst, Dr. Broadbent, Mr. Henry C. Burdett, and Mrs. William Black.

The report of the hon. secretary, Miss Honnor Morten, gave most satisfactory proof of the prosperity of the association. There are 204 nurses now on the roll, and 1,732 cases have been attended during the last twelvemonths. Every care is taken to secure and to retain the best nurses only. At 8, New Cavendish Street, a room has been set apart for sick nurses (non-infectious cases only), and the attendance of the hon. physician and surgeon are gratefully appreciated. It is computed that the average earnings of the nurses amount to £96 per annum, to quote from the printed report, "but of course one of the advantages of the Co-operation is that it gives to the kindly and competent nurse the chance of profiting by her popularity, allowing her to register her own fees, and to go to all cases where her services are specially requested." The speech of the late chairman, Mr. Cheston, was a particularly pleasant one, and Dr. Goodhart's witty description of the labours of an hon. treasurer occasioned much amusement. Mr. Slaughter gave details of the first establishment of the Co-operation, and in deploring Mr. Burdett's absence, he referred to the great interest he had taken in the scheme and to his share in its formation. All the speakers cordially agreed as to the admirable management and brilliant administrative abilities which the Lady Superintendent has displayed in bringing the association to its present prosperous condition. Not only does it pay its way, but it is hoped that the 7½ percentage which is all that the nurses now pay on their earnings for covering working expenses, may by and by be reduced as low as 5 per cent. The Home Sister's kindly and able fulfilment of her duties was heartily acknowledged, and equal justice was done to the unwearied exertions of the hon. secretary, who gave so much valuable assistance in conquering those initial difficulties which beset fresh undertakings.

### Appointments.

[It is requested that successful candidates will send a copy of their applications and testimonials, with date of election, to The Editor, The Lodge, Porchester Square, W.]

Miss Nott-Bower, the most courteous and able Lady Superintendent of Guy's Hospital Trained Nurses' Institute, has recently been promoted to the important post of Matron to the same hospital. Miss Nott-Bower commenced her training at Guy's in 1882, and was subsequently Matron for a short period of the Huddersfield Infirmary. She has already done much for the wellbeing and comfort of the nurses at Guy's Hospital, and her appointment as Miss Jones' successor is well calculated with Dr. Perry's co-operation to very materially improve the nursing arrangements there.

Miss H. Hadley Scott has been appointed Matron of St. Catherine's Hospital for Cancer, at Bradford. Miss Hadley Scott was trained at the General Hospital, Manchester, and was afterwards for five years a member of the Manchester Sick Poor and Private Nursing Institution. For one year she was on the nursing staff of the Victoria Home, Bournemouth, and then became staff nurse at the National Hospital, Queen Square. Miss Hadley Scott held this responsible post for nearly three years, and her departure is much regretted by the staff and by the patients, and her testimonials are excellent.

### The International Nursing Congress, Chicago.

WE received the following communication from Miss Isabel A. Hampton, chairman of the nursing section of the congress, after we had gone to press last week. It was so important, however, that we determined to give it in a second edition. May we venture to hope, and to urge once more, that all who have a claim to be regarded as amongst the leaders of nursing in this country will combine to make the first Nursing Congress a great success. If wise counsels prevail, the Chicago Congress, as a neutral centre, where all can meet on equal terms, may result in reconciling conflicting interests and views to the no small advantage of sound nursing all the world over.

"Dr. Hurd has shown me a telegram from Dr. Billings, in which you ask if I had appointed Mrs. Bedford Fenwick to organize the British section of the International Congress of Nurses. The reply was that I had not. Permit me also to explain that as the International Nursing Congress was first planned, Mrs. Fenwick was appointed by the Chicago Committee as chairman of the British section. After it became part of the section on hospitals, &c., it was not thought necessary to have the appointment continue. Consequently, I am arranging in England and all Great Britain for papers and discussions, and am writing to the people myself. In doing so I am not giving Mrs. Bedford Fenwick, the Nurses' Association, nor the Nurses Pension Fund members any special consideration, but am asking only for the co-operation of all British nurses themselves. Any unpleasant feelings or conditions that may exist among themselves will not, I trust, be brought in any way into the Congress, which should be of interest and benefit to all nurses. I write you this as you are the editor of a nursing journal, and I should be sorry to have any statements appear through misunderstanding."

### Everybody's Opinion.

*[Correspondence on all subjects is invited, but we cannot in any way be responsible for the opinions expressed by our correspondents. No communications can be entertained if the name and address of the correspondent is not given, or unless one side of the paper only be written on.]*

### INDIAN NURSING.

"NURSE F." writes: Seeing the letter "Tommy Atkins" writes to the *Times of India*, I would be glad to know if there is any society in England for supplying nurses for the outlying districts of India? I fully sympathise with the British soldier abroad, knowing from experience the need there is for nurses in those parts. Having spent a few years there myself, I would be very glad of any information on the subject.

### AMERICAN DIETS.

"A TRAINED NURSE" writes: Permit me to say a few words about patients' food in England as compared with America. In the latter country the meals are three in number, neither more nor less, both for patients and for nurses. In England the patients' meals in the best managed hospitals and infirmaries are always five, namely, breakfast, lunch, dinner, tea, supper. The English food may be of a lighter character, but the fact remains that the meals are five in number as compared with three in America. Two well-known American authorities on nursing were in England last year, and on visiting our general hospitals they invariably remarked, "The patients seem always in course of being fed over here!" They also spoke in great praise of the method of serving the meals in the wards, and the admirable way in which the food reached the patients as regarded appearance and method. Comparisons drawn between the two countries by experienced American workers may be considered at least as fair as those of an English visitor to America.

### WANTED, GOOD NURSES.

"A NURSE" writes: I became a poor-law infirmary nurse in answer to an advertisement, offering salary £25 with usual rations. On entering on my duties I found no beer allowed, nor its equivalent in money, nor uniform, which makes salary about £20. I have entire charge of seventy-seven beds, surgical and imbeciles. Now, the question I ask is, What thoroughly good trained nurse could possibly give time, skill, &c., for that salary? She may do it as a labour of love, and as unto the Lord; but many, like myself, may have an aged parent to help. If other guardians offered, like those at an infirmary in Kent, £30 per annum, and 5s. for every midwifery case, also beer and uniform, dozens of women would answer the advertisement instead of only one doing so. Every labourer is worthy of his hire.

"A CONSTANT READER" says: In a recent number of THE HOSPITAL it was asked "when will more of our best nurses be willing to utilise their skill and their experience for the benefit of patients in poor law infirmaries?" I think that if all infirmaries containing over 100 beds were placed under the authority of properly qualified people, such as medical officers and trained matrons, more good nurses would seek appointments in them.

NURSE W. asks: Will readers of THE HOSPITAL who have worked in infirmaries tell us something about the hours and general duties of nurses there? Is the help given by the convalescent inmates of much value? Is extra assistance granted to a charge nurse when all the patients happen to be bed-ridden? Are the probationers moved to different wards every three or six months? It would be interesting to have some reliable information on these and other points.

NURSE FANNY writes: It is well that THE HOSPITAL is taking up such a subject as "Wanted, Good Nurses." I think there is noble work to be done in workhouse infirmaries, and certainly it needs noble women to do it. Those ladies who want "sensations" and easily-earned "distinctions" cannot get their will in these places. Honest work, monotonous cases, and great responsibility are the portion of those who give their lives and their talents to the destitute and hopeless poor. Good women are needed for this work, certainly not the girl-nurses of whom "An Experienced Nurse" wrote so truthfully. I should like to thank her for her letter.

### GIRL-NURSES AND INFIRMARIES.

"A THOUGHTFUL WOMAN" says: I am pleased at what your correspondent "Experienced Nurse" says about girl nurses. The subject goes hand in hand with workhouse nursing. It is terrible to think of young women of twenty in such wards; the work is most improper for them in every way. Why don't the Guardians spend more money in wages for qualified nurses? They try and get the most important work done cheap, I suppose. They build most beautiful infirmaries, and give rooms and food to patients and nurses of which the staff of general hospitals may well feel envious, but the supply of workers is most inadequate. The number of patients to each nurse is truly appalling. If a Guardian would become an inmate for twenty-four hours at the worst season of the year he would buy an amount of personal experience which would stand him in good stead and make him, indeed, a wiser, if a sadder man.

### MRS. WARDROPER.

E. BISHOP writes: I enclose one shilling as subscription towards the proposed memorial to the late Mrs. Wardroper. All nurses ought to feel grateful to her for the benefit she conferred on us by raising the tone of nursing and making it the valuable occupation for women that it now is. I also send one shilling towards the Disabled Nurse Fund.

### A DISABLED NURSE.

Sister Moira writes: Please add enclosed small sum (five shillings) to the subscription for our disabled sister. Surely we should thank you for bringing her sad case forward and allowing us the privilege of helping each other.

"A Disabled Nurse" writes: Please accept most grateful thanks for postal orders, and also for kindness in making this collection. May I ask you to thank all the nurses for their most practical sympathy. The help will be most acceptable, as this long illness has been a very great expense, and it seems likely to go on for some time.

### "THE HOSPITAL" ENDOWED BED.

Rose M. Minty writes: I shall be pleased to collect £1 and upwards.

Nurse Perry and Nurse Colebrooke each offer to collect £1 towards Endowed Bed.

### Croupous Pneumonia.

Paper read before the Nurses' Journal Club at the Training School for Nurses of the Johns Hopkins Hospital, April, 1892.

There is no disease more interesting to nurses than pneumonia. It has long been acknowledged one in which nursing is more important than drugs. Short, acute, of marked character, and running a definite course, it gives the nurse opportunity to exert every power she possesses.

Its outward aspect and characteristic symptoms are all perfectly familiar. We knew them all long ago, but knew nothing of what they meant or what was going on within. The inner history of the disease was hidden.

As one watches the actions of one's friends without understanding their motives, or knows history by dates and statistics only, the hidden mainsprings of action unsuspected, so here one saw only results, not the cause.

If interesting then, how infinitely more so now with the light of modern research thrown upon it. It has been my great good fortune this winter to hear a series of lectures by Dr. William H. Welch considering pneumonia from the standpoint of the bacteriologist; lectures profound and exhaustive, and more fascinating than romance. I shall try to put together in this paper, though necessarily in a fragmentary way, some gleanings from the wide field of those lectures by sketching an outline of the bacteriological history of a typical case of acute croupous pneumonia ending in recovery.

Pneumonia is now scientifically classed among infectious diseases because it is produced by a specific micro-organism. Contagious it cannot properly be called. This rather vague term is best restricted to those diseases which are communicated with great readiness from one being to another. The degree of readiness depends upon the way in which the germs leave the body. Scarlet fever is an example of an infectious disease which may also be called contagious. Here the germs are in the skin, and can with the greatest facility be transmitted from sick to well persons. Indeed, it would be almost impossible to prevent this. At the other extreme is malaria, an infectious disease which is never contagious, because here the organisms do not leave the body at all. Midway stand such diseases as typhoid fever, phthisis, and pneumonia, in which the germs leave the body by certain definite routes—the stools, the sputa—and while infection may occur, yet with care it is perfectly possible to guard against it without isolation of the patient.

The organism of pneumonia is on the border line between a bacillus and a micrococcus. It would seem to belong to the royal family of germs, for it has six and even more names, most of them double. Of these, the Pneumococcus of Frænkel and Weichselbaum, Micrococcus Pasteuri, Micrococcus of Sputum Septicæmiæ, Micrococcus Pneumoniæ Crouposæ, are the most unwieldly; Pneumonococcus the most convenient, though etymologically not correct; Diplococcus Pneumoniæ and Diplococcus Lanciolatus the most dignified and appropriate.

The history of its discovery is interesting. Friedländer, in 1883, the pioneer, meant to find it, and thought he had done so; described carefully an organism (now called the Pneumobacillus of Friedländer) but described the wrong one. Sternberg, in 1880, found the true pneumonia coccus in his saliva, but he did not know it as such, and afterwards identified with it Friedländer's bacillus. Pasteur, in January, 1881, published the first description of it. Frænkel and Weichselbaum in 1885 and 1886 fully described the organism and demonstrated its relation to pneumonia. Their work is regarded as the fundamental one on the subject.

In experimental work it is found to vary greatly in virulence. It grows best at a temperature of about 98 F., and is both aërobic and anaërobic; that it can live with or without oxygen.

Cultivated artificially its life-history bears a curious resemblance to the rise and fall of nations. At first, strong in its native quality of virulence, it refuses cultivation, and its growth on artificial media is weak and feeble, but with successive generations it grows more and more luxuriantly, and loses its power.

Its pathogenic effect varies. Two factors are present to modify its course, viz.: its own degree of virulence and the degree of susceptibility of the creature exposed. Human beings are among the relatively insusceptible.

Locally it may be a pus producer. It will form abscesses in human beings and is also capable of invading the blood and producing many widely different results.

(*To be continued*).

### Notes and Queries.

SPECIAL NOTICE.

The contents of the Editor's Letter-box have now reached such unwieldy proportions that it has become necessary to establish a hard and fast rule regarding Answers to Correspondents. In future, all questions requiring replies will continue to be answered in this column without any fee. If an answer is required by letter, a fee of half-a-crown must be enclosed with the note containing the enquiry. We are always pleased to help our numerous correspondents to the fullest extent, and we can trust them to sympathise in the overwhelming amount of writing which makes the new rules a necessity.

### Queries.

(32) *Examination Questions.*—Are all your readers free to answer these?—*Warwick.*

(33) *Convalescent Letter Wanted.*—Will anyone kindly give a poor woman (Churchwoman) a free letter for a convalescent home?—*M. D. Churchill.*

(34) *Note Book.*—Can you tell me of a suitable note book for private nursing?—*Carlisle.*

(35) *Physiology.*—Can you recommend someone who coaches in physiology, either in classes or single pupils?—*Nurse Alice.*

(36) *Probationers.*—Will you kindly write me particulars as to training?—*Laburnham.*

(37) *District Work.*—Where can I get training in district work?—*Nurse C.*

(38) *Chronic Colotomy.*—Can anyone tell me of a home in London or provinces where such a case can be received at moderate terms?—*A Friend.*

### Answers.

(32) *Examination Questions* (Warwick).—Certainly.

(34) *Note Book* (*Carlisle*).—The "Nurse's Case Book," price 6d., from "Scientific Press" Office, 140, Strand, W.C.

(35) *Physiology* (*Nurse Alice*).—Do you mean elementary physiology only, and do you wish for a lady teacher?

(36) *Probationers* (*Laburnham*).—You will find all particulars in "How to Become a Hospital Nurse and How to Succeed," by Honnor Morten, price 2s. 6d., 140, Strand.

(37) *District Work* (*Nurse C.*).—Apply for information to Superintendent, Q.V.J.I., at St. Catherine's Hospital, Regent's Park.

(38) *Chronic Colotomy* (*A Friend*).—We advise your advertising in The Hospital for a Home for Incurables where there are trained nurses. See list in "Burdett's Hospital Annual," new edition.

### The History of Nursing.

Notes of an introductory lecture given by Dr. SHAW to the nurses of the Bristol Royal Infirmary.

MOSES educated in medicine in the priest-schools of Egypt (Clement of Alexandria); in his sanitary code makes no provision for treatment and care of the sick, unless for the skin diseases included under the term leprosy.

Israelitish women educated in midwifery in Egypt.

Scarcely any allusion to sick-nursing in the Old Testament. Bodily sickness considered to be a punishment from God, to be treated by prayer and the intercession of Priest or prophet.

Pliny in his "Natural History" remarks that Homer says that for the relief of sickness prayer must be made to the gods.

St. James in his General Epistle recommends the same procedure, along with laying-on of hands and anointing with oil (chap. v. ver. 14, 15).

Systematic nursing of the sick apparently instituted by St. Paul and St. Luke. The latter, a Gentile, born at Antioch (Eusebius), and educated as a physician ("Luke the beloved physician") probably at the neighbouring medical school of Tarsus. Close to Tarsus was a celebrated shrine of Æsculapius, whither the sick of the neighbourhood would be brought for advice, which would correspond to the hospital of a modern medical school; here, perhaps, both SS. Paul and Luke gained professional experience and practice.

A.D. 55—Phœbe, Tabitha (or Dorcas), and others appointed to visit the sick in the Christian community in Jerusalem and elsewhere, visiting from house to house. These women were called servants of the Church, or "deaconesses."

Somewhat later Christian communities were formed in many cities; in each seven elders (presbyters) were appointed, who gave the collected money to deacons; these male servants of the Church sought out and nursed (apparently) the sick men, while the girls and women called deaconesses still cared for the sick women. All these services were unpaid and performed voluntarily.

As the communities continued to develop, the elders appointed overseers (Bishops) in the larger communities, who left the personal care of the poor to the deacons, who in turn compelled widows to perform the requisite nursing and education. These widows were supported by the community. The widows failed to attend properly to these duties. Therefore, in

A.D. 300, in the eastern half of the Roman Empire, widows were again replaced by deaconesses. These deaconesses wore a veil as a mark of distinction, and were consecrated for their work by laying on of hands by the Bishop. Owing to the poverty and persecuted condition of the Christians only "district nursing," so to speak, was possible still.

A.D. 330.—Christianity declared by the Emperor Constantine to be the State religion of the Roman Empire. Community nursing gave place to nursing in special charitable institutions. These originally were rooms near the Bishop's house, and were open to all in poverty or want; the nursing, as formerly, being managed by deacons and deaconesses. The number of sick applying for relief soon made it necessary to build hospitals proper, as at Edessa (200 beds), Alexandria, &c. The funds of these institutions were managed by the Bishop, who appointed the officials, the Grecian doctors, and the persons employed as nurses.

During the 4th century deacons and deaconesses became fewer and were replaced by hired attendants, who subsequently became insubordinate and unsatisfactory. St. Jerome relates that the first Christian hospital in Rome was founded by Fabiola, a patrician widow, at the end of the fourth century. Other rich persons followed her example. The Church pronounced the care of the sick pleasing to God—Fabiola "carried the sick to their beds in her arms, and washed out wounds which others dared scarcely look upon" (St. Jerome, Ep., 84). The Empress Placilla Augusta undertook the duties of a maid-servant in the hospitals. In the eastern half of the Empire it is similarly recorded that ladies sought in nursing, "a cure for melancholy," as Olympias, the 18 year old widow of the Prefect of Constantinople, and Marcina, the sister of a Bishop, whose betrothed had died. At the Hospital of St. Sophia, in Constantinople, in the fifth century it is recorded that 100 deacons and 50 deaconesses were employed in nursing.

A.D., 600.—Owing to the conduct of the paid nurses (ministri), Pope Gregory commanded that only "Religiosi," *i.e.* monks or nuns, should be entrusted to nurse in charitable institutions, but noble Roman ladies continued to act in menial capacities, as bed makers, &c. Eastern half of Roman Empire conquered by Mahomedans in sixth and seventh centuries; nursing therefore ceased in that part of Europe and Asia.

(*To be continued.*)

### For Reading to the Sick.

TRAINING.

THOSE who have accomplished any great work in life, have generally had periods of long and special training, and this is what has enabled them, when their opportunity came, to succeed where others failed. We are very apt to forget this, for whenever we see a successful man we think he was born under a lucky star, or born with wonderful talents, far greater than our own, for instance. Perhaps he may have more than ordinary ability, but that is not all, for that which we call genius and which seems to burst suddenly on a dazzled world, is nought but great talents joined to unwearied perseverance; hours and days and years of hard work have been the careful preparation for what we all admire or envy. The great singer or musician who charms us by a delightful performance, which lasts but a few minutes, has won the applause of thousands by the unremitting toil of years. Painter, preacher, orator, surgeon, one and all have worked at their particular calling with an industry which will not be daunted. And though they can never satisfy themselves, yet their aim being single, they love to learn more and more of that which will benefit or instruct or please their fellow men.

And this training is as necessary for spiritual as for mental and bodily perfection. When God raised up anyone for a special work, their preparation was long and careful and severe. Moses, St. John Baptist, St. Paul, are names which occur to us in this connection, and often the period of work time seems very disproportionate to that of training; a few years at most, to practice what it took a lifetime to learn, but which left its impression upon centuries.

Our Lord's public life lasted but little over three years, while His hidden life at Nazareth occupied thirty, during which He was preparing for that brief but bitter agony which was to influence all time.

Shall we then grudge the toil, the sorrow, the pain which is to prepare us for a glorious eternity? But we are often unwilling to strive to work out our salvation, that is, do our own part in unison with God, so our loving Father keeps our hands to the plough, though we from time to time turn our heads back. We are wanting in patience, and we learn it by being tied for months to a sick-bed; or we fail in humility, God sends us checks and snubs in life, and misfortunes till we cease to "exercise ourselves in great matters," till we can say with the Psalmist "My soul is even as a weaned child," while the fortitude which calmly awaits whatever may befall us is only reached by a constant dependence on our Master's will. This perfection which God requires, and which man admires, must like everything else, be but the result of training and moulding ourselves by the glorious Pattern we have ever set before us. Like Him we will devote our lives to preparing for times of adversity and trial, and welcome our sickness and our chastenings as the means by which we shall become meet for His Kingdom and shine like stars in the firmament of Heaven.

### Some Australian Experiences.

(*Continued from page* xciv.)

### MY RECEPTION.

At length we saw lights, and I was told this was the "Golden Gate." The first "claim" passed, then we were on "Croydon." It was so late that the town was in total darkness, except the hotels; and as we drove up to the "Post Office" a voice out of the darkness asked, "Is Miss —— on the coach?" I answered, "Yes." Then said the voice, "I am here to meet you." I gladly alighted, and found a white-haired old man, who informed me he was the secretary of the hospital, where he would conduct me if I liked. I said, "Get me a glass of clean water before you do anything"; so he brought it out of the darkness. Then we walked to the end of a street, where far up on a sort of rising ground was a bright light. "That is the hospital light," said he—a most welcome sight, too. We walked up, and I was shown into a nice clean kitchen, where a sort of tea was laid. "This is curious," thought I, but said nothing. Two clean servants came to assist me off with my dusty things, and took me into a sort of linen closet, with a bed, a table, and chair. This was my bed-room. The nice white bed looked very inviting, so after a wash and some refreshment I retired. That was all I saw of the hospital that night. There were only six patients, all just asleep; and not needing anything till morning, utterly worn out I soon slept, and was surprised to find myself so fresh in the morning. When I arose and looked out, I was most pleasantly surprised to find the place surrounded by hills. Arriving in the dark I had not seen the pretty range of hills near the "Golden Gate." This was cheering after the many miles and miles of flat country we had traversed.

I dressed, and went on a tour of inspection round the "hospital," not unaware of the fact that I also was being inspected. I think the patients got up a little earlier to see "our new matron," and this is what I found: A nice ward (male); 12 beds, a locker beside each, but no table down the centre (the width of the ward would not permit it); each bed was nicely fitted with a wire spring mattress and clothes; mosquito-net and white counterpane; each patient was supplied with a set of pyjamas by the hospital; a broad verandah ran round the ward, where there were six more bedsteads to put the bad cases out on in the day-time, or for when the hospital was full. Like all Queensland houses, &c., the hospital was built on piles; ours were four feet high, making a good current of air underneath. A small bridge separates the large ward from the living rooms, across which was first the kitchen before mentioned, then my bed-room, servants' ditto, round the corner my sitting-room, beyond that a perfect gem of a dispensary, where there was everything for use. This was Dr. Flood's pride. He got the whole thing up himself, and all the drugs from England (Ferris and Co.). Across another bridge was what was supposed to be the women's ward, a wooden frame with white calico stretched over it, like a huge Punch and Judy show. I had the pleasure of getting the whole thing reconstructed, and a very comfortable little ward made of it before I had been there long. Until it was done it was not fit to nurse in, the heat was so terrific. The hospital being on a ridge, we looked straight into the town, and very curious it looked with its corrugated iron, calico, canvas, and wooden houses, and tents dotted about in every direction; then the shafts of the different mines, and the thumping of the crushing machine, all impressed you with the fact that the place was a busy one.

My first caller was a deputy from the committee keen on retrenchment (the order of the day in Queensland). From an allowance of three, I was requested to try with two servants. After a mutual quizzing, he departed.

There were no ladies on the "field" at this time, all the men of social position being single; so the usual order of things was reversed, and I had a perfect army of gentlemen callers, very kindly disposed, too, as they afterwards sent up books and papers for my amusement.

Everyone looked so nice and cool in their white clothes, and extremely dressy too, with prettily-figured skirts, or cream silk ones, with a bright-coloured cummerband, and either a white helmet or grey soft felt hat, with silk pugaree round. The heat was very great just then (September), 120 to 130 deg. F. in the shade, water getting more scarce each day. (I mentioned before there had been no rain for two years). Every particle of grass burnt up, and the poor bushes shrivelled up. At no time is the foliage very luxuriant in the "Gulf Country," as it is called, the soil being so very poor. After the rainy season it is at its best for about two or three months, and the appearance of the place is totally altered.

Of course, fruit or vegetables had not been seen or heard of for months, saving tinned peas. Fresh milk and butter were as some dream in the far distance—everything was tinned, tinned, tinned; and such a price, too, regarded as luxuries, the general fare being salt beef and damper (a sort of soda cake). With this hard fare and bad water it was no wonder that the "gulf fever" set in earlier than usual, and filled up the hospital inside and outside. On the top of this came dysentery and one case of typhoid; then came the tug of war. Nursing *was* nursing then with a vengeance. It is one thing to tussle with typhoid and dysentry in a town hospital—where you get fresh milk, eggs, and all sorts of medical comforts—but quite another to do it on stale condensed milk and second-rate whiskey, for that was the range of my diet scale. Until then the "local fever" (which is peculiar in its character, the temperature rising between ten a.m. and four p.m., and, unless a very severe case, down again at sunset till next morning) had been treated with "Warburgs's Tincture," which certainly reduced the temperature in a remarkable manner, but also the patient, to such a degree that he often never rose again. So I consulted with the doctor, who very kindly allowed me to try sponging, which I continued with great success all through the summer, and at the end of my first year there my death-rate stood 9, against 28 of the year previous, with only a difference of four cases admitted.

My sponging was novel, as there was no such thing as a mackintosh or sponge in the place, so I placed a bedstead with a wire mattress on the sheltered side of the verandah, put a blanket and pillow on it; on this I laid my patient with nothing but a sheet over him; I then took a soft towel and begun by squeezing tepid water all over his head and gradually the length of his body, then when he grew accustomed to this I used cold water, then put him back to bed in blankets, the usual result being a remission of temperature, perspiration, and sleep, from which he awoke greatly refreshed. I have admitted men with temperatures of 106, and in two days got them normal by this treatment and plenty of whiskey. The usual period of the "gulf fever" is three weeks in an acute form, but one can have it for months at a stretch, at one time feeling well, then bad again, alternately. When this condition occurs, nothing but a complete change of air from the district will effect a cure.

The complications are "Bright's" severe dyspepsia, or the "Fever Ball" as the men call it. They describe the pain as "like a ball pressing on the sternum." Enlarged glands in the groin and axilla, which vary a good deal, sometimes disappearing as the patient's condition improves, or pointing and requiring to be opened. The men call these "gulf lumps."

(*To be continued.*)

### Presentations.

Dr. T. Laurie Gentler, L.R.C.P.Lond., was recently presented with a handsome gold albert chain and illuminated address in recognition of his services to the nursing staff of the Derby and Derbyshire Royal Nursing and Sanitary Institute.

